# Does working memory protect against auditory distraction in older adults?

**DOI:** 10.1186/s12877-020-01909-w

**Published:** 2020-11-30

**Authors:** Yatin Mahajan, Jeesun Kim, Chris Davis

**Affiliations:** 1 The MARCS Institute for Brain, Behaviour and Development, Western Sydney University, Penrith, New South Wales Australia; 2 The HEARing Cooperative Research Centre, Melbourne, Victoria Australia

**Keywords:** Distraction, Older adults, Working memory, ERP

## Abstract

**Background:**

Past research indicates that when younger adults are engaged in a visual working memory task, they are less distracted by novel auditory stimuli than when engaged in a visual task that does not require working memory. The current study aimed to determine whether working memory affords the same protection to older adults.

**Method:**

We examined behavioral and EEG responses in 16 younger and 16 older adults to distractor sounds when the listeners performed two visual tasks; one that required working memory (W1) and the other that did not (W0). Auditory distractors were presented in an oddball paradigm, participants were exposed to either standard tones (600 Hz: 80%) or various novel environmental sounds (20%).

**Results:**

It was found that: 1) when presented with novel vs standard sounds, older adults had faster correct response times in the W1 visual task than in the W0 task, indicating that they were less distracted by the novel sound; there was no difference in error rates. Younger adults did not show a task effect for correct response times but made slightly more errors when a novel sound was presented in the W1 task compared to the W0 task. 2) In older adults (but not the younger adults), the amplitude of N1 was smaller in the W1 condition compared to the W0 condition. 3) The working memory manipulation had no effect on MMN amplitude in older adults. 4) For the W1 compared to W0 task, the amplitude of P3a was attenuated for the older adults but not for the younger adults.

**Conclusions:**

These results suggest that during the working memory manipulation older adults were able to engage working memory to reduce the processing of task-irrelevant sounds.

## Background

The occurrence of an unexpected sound when performing a visual task (e.g., determining if two digits are the same or not) can be distracting. Although distraction may affect task performance, processing distractors is important for being aware of potentially important events outside the task domain.

The extent to which a distractor is processed and attended depends upon how it fits with an on-going model of the environment, i.e., when a distractor is novel it is more distracting [1]. Also, distraction can be modulated by the degree to which a task is attended [2, 3]. In this regard, it has been proposed that working memory (WM) acts to enhance and maintain the sensory processing required by a task, while at the same time reducing processing triggered by the presentation of a distracting stimulus [3–6].

Evidence from younger adults that engaging WM reduces distractor processing and distraction comes from several studies [3, 7]. For example, the SanMiguel et al. [3] study used a cross-modal distraction paradigm in which participants performed either a working memory (W1) or no working memory (W0) visual matching task while auditory distractors were presented. It was found that the presentation of novel distractor sounds slowed responses in the W0 condition but not in the W1 condition. That is, there was less distraction in the WM task.

In addition to using behavioural measures of distraction (i.e., hit rate and response time), event-related potentials (ERPs) can also be used to determine the impact of working memory on the neural indices of distraction. ERPs provide a method to assess the brain function in response to sounds that has two major advantages compared to other neuroimaging procedures: excellent temporal resolution (in the order of milliseconds) and cost-effectiveness. The high temporal resolution ability of the ERPs have been used to investigate, low-level cognitive functions such as encoding of sounds [8, 9], high-level functions such as selective attention, working memory, and language [3, 10, 11] and functions intermediate between low and high cognitive functions such as sound discrimination, and involuntary attention [12–14]. The ERPs and behavioural measures index different information regarding cognitive processing. While, ERPs provide processing information before, during and after a cognitive response, behavioural measures are usually related to the processing after the response.

Information processing related to distraction has been described by a model that posits three sequential processing stages with each stage reflected by a specific event-related potential (ERP), see Horváth, Winkler and Bendixen [15]. According to this model, the initial stage of processing acts as a filter which adaptively adjusts the processing of sensory information to reduce the load on capacity-limited resources. This change detection stage is often associated with the mismatch negativity (MMN) ERP, a negative wave resulting from the subtraction of the ERP to a standard stimulus from that of a deviant one that peaks between 100 and 200 ms from the onset of the detected deviance. The MMN reflects a largely memory-based process that detects deviations from perceived regularities in the auditory input [16]. There is, however a more basic process that is sensitive to stimulus onset and simple change detection, which is reflected by the N1 ERP, a negative potential that peaks fronto-centrally between 100 and 150 ms following a stimulus change [17]. Given the similarity of the functions represented by the two event related components (to filter and highlight sensory events), it is often reported that this first stage of processing is represented jointly by N1/MNN, although Horváth et al. [15] take care to separate these, which we do in our analysis.

The second stage in the model of distraction deals with the process of involuntary attention-switching mechanism towards the distractor once it has been detected as a change from some regular sensory context. Here, cognitive resources are allocated between voluntary attention to task-relevant events and involuntary attention to distractor events. This stage is marked by the P3a, that peaks between 200 and 300 ms after the distracting event [18]. It has been suggested that the P3a represents an involuntary change in selective attention set that is invoked by the distracting event [19]. That is, it is a cognitive orienting response that typically is generated to rare stimuli and may also be associated with arousal [20].

The function of the third processing stage of the distractor model is to switch or refocus attentional resources back to task-relevant events [21]. That is, provided the event that triggered a switch in attention does not require an on-going adaptive reorientation, the original task-related attentional set is restored. It has been proposed that this function is reflected by a modality-independent, fronto-central reorienting negativity (RON) component [13, 22].

In their study, SanMiguel et al. [3] found that for the working W1 task (as compared to the W0 task) there was no change in MMN amplitude; there was a reduction in the novelty-P3 amplitude (the P3a), and a larger RON in younger adults. The current study aimed to investigate the effects of working memory load on both the behavioural and neural indices of distraction processing in older adults.

There is a large and inconsistent research literature that has used both behavioural and ERP measures to examine age-related changes in auditory distraction. For example, some behavioural studies report that older adults’ control of auditory distraction is impaired [23], while other studies find equivalent distraction levels between younger and older adults [24, 25]. The ERP literature is similarly mixed. For example, in terms of age-related differences on MMN amplitude some studies report age-related reductions in MMN amplitude in response to deviant tones, suggesting older adults have deficits in encoding and retaining sensory information, reducing their ability to detect distractors within encoded sequences [26, 27]. However, others find no age-related differences on MMN amplitudes or latencies, suggesting that older adults do not have deficits in automatic processing (involuntary, early attention) [28, 29]. Research that has examined the later ERP components, P3a and RON has tended to paint a more consistent picture concerning age differences (although see Berti et al. [30]). For example, it has generally been found that older adults have a smaller [26, 31], and a later [29] P3a compared to younger adults. This has been interpreted as indicating that older adults do not evaluate auditory distractors as efficiently as younger adults [32]. Likewise, studies have indicated that the amplitude of the RON is also smaller (and occurs later) in older compared to younger adults, a finding suggesting that older adults are less efficient in re-focusing their attention to the task following distraction [30, 32].

The different behavioural and ERP results outlined above highlight the difficulty in drawing conclusions from studies that have employed diverse experimental paradigms. In the current study we chose to extend the classic procedure used by SanMiguel et al. [3] by examining the extent to which older adults (62–74 years) are protected from distraction when engaged in a working memory task (for comparison, younger adults aged 22–35 years were also tested). We selected the SanMiguel et al. procedure since it produced clear behavioural and ERP effects; used a simple cross-modal distraction paradigm and employed a categorical contrast between a working memory task and a similar task that did not involve working memory. In our view, when aiming to examine perceptual and cognitive effects on a different participant group it is an important first step to build on earlier studies by using their procedures.

SanMiguel et al. [3] used an auditory oddball paradigm to determine the impact of working memory on the neural indices of distraction. The current study used the same paradigm to elicit neural indices of distraction processing in older and younger adults. In an oddball paradigm distractor sounds are the infrequent stimuli interspersed randomly but with a pre-determined probability in a series of repetitive frequent standard sounds. Before outlining the details of the current study, it is important to emphasize that although the results of SanMigel et al. [3] clearly supported the claim that engaging working memory reduces the effects of a distractor, other studies found the opposite pattern, i.e., the involvement of working memory increased distraction [33]. There are several reasons why engaging working memory may not always reduce the effect of a distractor and in the following section we outline some of these.

One factor that may influence the effect of working memory on distraction has to do with the relationship between the distractor and the task stimuli. For tasks where target and distractor stimuli compete for sensory processing and response selection (e.g. a unimodal task and distractors), increasing the working memory load tends to increase distraction [34]. Whereas, for tasks where the distractors do not trigger response competition, engaging working memory typically reduces distraction [3, 5].

Another factor that can modulate how working memory affects distraction concerns whether working memory is engaged as part of the task, or whether it is engaged in an unrelated task. That is, paradigms that load working memory with materials unrelated to the task more often report that working memory load increases distraction [34].

Finally, there is the factor of how much working memory load is applied. That is, it appears that whether distraction can be suppressed in the early stages of processing or not depends on the resources available for executive cognitive control [33]. The behavioural and ERP studies of SanMigel et al. [3] and Lv et al. [5] provide an example. In SanMigel et al., the working memory condition consisted of a simple visual 1-back task and performance in this condition was compared to that on a 0-back task, i.e., the contrast was with a non-working memory task. Two types of auditory distractors were used, a 600 Hz tone that occurred 80% of the time before the visual task (the standard); and novel environmental sounds that occurred 20% of the time. Overall, the presentation of novel compared to standard distractors reduced the hit rate and increased the response times to the visual task. This effect was driven by performance in the no working memory condition (0-back task), since in the working memory task there was no effect of distractor type (i.e., engaging working memory protected against distraction from the novel sounds). As mentioned above, SanMigel et al. [3] also found that the novelty-P3 was attenuated in the working memory condition.

For the most part, this effect of working memory protecting from distraction was not found by Lv et al. [5] who used the same cross-modal distraction paradigm but contrasted an easy and a difficult working memory task. That is, unlike SanMigel et al., Lv et al. found that engaging working memory (in their case, the high versus low memory condition) did not protect against being slower and less accurate on the visual task when presented with novel compared with standard distractors. Also, unlike SanMigel et al. who found no effect, Lv and colleagues found that an early ERP component (MMN) had a greater amplitude in the high load condition, suggesting that high memory load increased the salience of the novel distractor. Finally, and similar to SanMigel et al., Lv and colleagues did find later processing of the novel distractor (as indexed by P3a) was attenuated in the high WM versus the low WM condition. Similar findings also have been reported in auditory-only tasks. Evidence suggests that the amplitude of P3a and RON ERPs decreased in younger adults with increasing load in tasks, when WM load was modulated within the auditory domain only [4, 35, 36]. On the other hand, the stage of distractor processing indexed by MMN, appears to be minimally modulated by task demands [36] in younger adults.

As the current experiment aimed to determining whether engaging working memory will reduce distraction in older adults, we chose to use the cross-modal distraction paradigm employed by SanMiguel et al. that contrasted a working memory and a no working memory condition, since this design has produced the clearest effect of working memory on distraction in both the behavioral and ERP data. It is, however, an open question as to whether older adults will exhibit the same behavioral and neural effects of engaging working memory that younger adults showed in SanMiguel et al.

That is, two properties associated with cognitive aging could limit the extent to which working memory may protect against distraction. The first, is that older adults tend to be more distractible than younger adults, especially with cross-modal distraction (i.e., a visual task with an auditory distractor), see Leiva et al. [37], and more generally the literature on the inhibitory-deficit hypothesis [27, 38–41]. Older adults are more distractible than their younger counterparts in response to unexpected auditory events when engaged in a task that requires voluntary attention [42]. Research using cross-modal (auditory-visual) oddball paradigms where participants engaged in a visual task while ignoring auditory distractors, found longer reaction times and low task accuracy in older adults compared to younger adults indicating increased distractibility [23, 43]. Indeed, effects of increased distractibility in older adults have also been found in neural markers of distractor processing. Alain and Woods [44] found that while there were no age-related changes in visual discrimination task performance following auditory distractors, older adults had a larger N1 and a smaller MMN to auditory distractors. These results were interpreted as age-related decline in the ability to suppress irrelevant auditory stimuli supporting inhibitory-deficits hypothesis. Previous research using unimodal auditory oddball paradigms have also reported age-related distraction effects either on behavioural or neural indices. Accuracy of an auditory duration discrimination task was reduced in middle-aged adults in the presence of irrelevant sounds, but no effect of age was found for reaction times [29]. However, delayed P3a and RON latencies in middle aged adults indicated distractibility [29]. Age-related greater behavioural distraction effects in older adults and low-performing older adults compared to younger adults were found in similar auditory duration discrimination tasks [30, 38]. In a similar task, Horvath et al. [34] reported delayed P3a and RON ERPs in older adults but no age-related behavioural distraction effects. There is, however, a dearth of research examining ageing effects on auditory distraction using auditory-visual paradigms.

The second property associated with cognitive ageing is that older adults may be less able to engage sufficient working memory resources overcome distraction due to age-related deterioration in the functioning of dorsolateral pre-frontal cortex [45–48].

## Method

### Ethics

The research was approved by the human research ethics committee at the Western Sydney University. Written informed consent was obtained from each participant prior to the experiment.

### Participants

Sixteen older adults (OA, Mean age: 69.4 years, age-range 62–74 years, 7 males) and 16 younger adults (YA, Mean age: 26.4 years, age-range 22–35 years, 9 males) participated in this experiment. The older adults had normal hearing in both ears with hearing thresholds of ≤25 dB HL at 250 Hz, 500 Hz, 1000 Hz, 2000 Hz and 4000 Hz determined by the air conduction screening audiometry. The current sample size of 16 older participants will reliably (with probability greater than 0.8) detect effect sizes of δ ≥ 0.65 (an effect size that Cohen [49], classed as a medium to large effect size), if we assume a one-sided criterion for detection that allows for a maximum Type I error rate of α ± =0.05 (calculated with the JPower package in Jamovi, 1.2). Participants did not report any significant psychological or neurological history and had normal or corrected visual acuity. Older and younger adults were assessed on Mini-Cog test [50] comprising of a three-item recall and a clock drawing test to determine the cognitive status. No participant showed any indication of cognitive impairment (OA, M = 4.5; YA, M = 5).

### Experimental stimuli

**Fig. 1:**
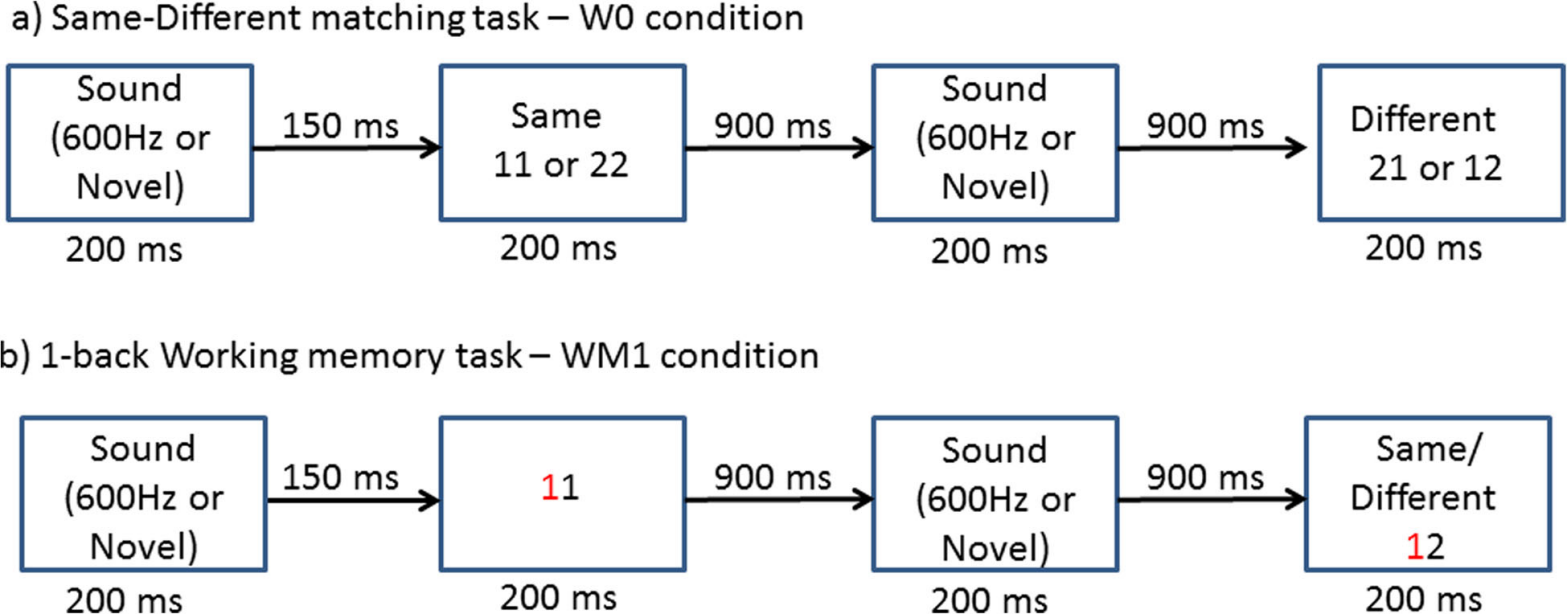
A schematic representation of two trials; a no working memory (W0) trial (upper panel) and working memory (W1) trial (lower panel)

Current experiment followed the experimental paradigm used by SanMiguel et al. [3]. It consisted of four blocks of 250 auditory and visual stimuli pairs with each pair presented at a fixed trial duration of 1250 ms. Each trial consisted of a task-irrelevant auditory stimulus (distractor) that was followed by a visual stimulus with a SOA (stimulus onset asynchrony) of 350 ms (see Fig. 1). Both auditory and visual stimuli were 200 ms in duration. Auditory stimuli were presented in an oddball design with frequent repetitions (80%) of 600-Hz pure tone of 200 ms (‘standard distractors’) and infrequent repetitions (20%) of 100 different novel sounds (‘novel distractors’). The novel sounds consisted of various environmental sounds such as animal and bird noises, a doorbell ringing, a hammer clanging, a sneeze, a cough, etc. and were selected from a database created by Escera et al. [51]. All sounds were digitally sampled at 44100-Hz with 10 ms rise and fall times and were presented binaurally via insert earphones at the most comfortable listening level for each participant, 75–80 dB SPL (sound pressure level). No novel stimulus was repeated within a block and each was only presented two times in the experiment. The sequence of oddball presentation was randomized such that no two novels were presented together and there were at least two standards before a novel sound was presented. The visual stimulus consisted of four pairs of digits (11, 12, 21 & 22), presented on a computer screen for 200 ms placed 1 m away from the participant. The presentation of digit pair was counterbalanced with each pair presented equally across four blocks.

### Experimental procedure

The experiment was carried out in an acoustically and electrically shielded Faraday cage. The experimental task required participants to attend to the visual stimulus and to ignore preceding irrelevant auditory stimulus. Participants completed two different conditions that varied in whether working memory was required (see Fig. 1). In the no working memory condition (W0), participants judged if two digits of the visually displayed stimulus were same or different. That is, pairs ‘11’ and ‘22’ should be classified as same, and pairs ‘12’ and ‘21’ as different.

In the working memory condition (W1), participants performed a 1-back working memory task. Here, participants matched the left digit of a visual stimulus pair on screen with the left digit of previous pair. That is, participants were required to remember the left digit and match this with left-most digit of the subsequent pair. For example, the correct response for a visual presentation of ‘11’ followed by a pair of ‘21’ should be different and ‘12’ followed by ‘11’ should be same. The buttons assigned for same and different responses were counterbalanced between participants. Participants had a maximum of 900 ms to respond to the visual task and no minimal time was specified. Keystrokes before the onset of the digits were not registered. Participants completed two blocks each of every condition and the order of blocks were counterbalanced as well.

### Electroencephalography recording

While participants performed each task, concurrent raw electroencephalograph (EEG) was recorded. Prior to fitting the electrode cap, scalp of each participant was prepared to reduce the time taken to achieve optimal scalp electrode impedance [52]. The raw EEG was recorded with a BioSemi Active-Two amplifier system (BioSemi, Amsterdam, Netherlands). Sixty-four Ag-AgCl electrodes were mounted on a nylon electrode cap according to the international standard 10–10 system [53]. There were two electrodes on the electrode cap that are unique to BioSemi system which served as online references [54]. Six additional electrodes were also placed on the participants; four were bipolar electrodes placed above and below the left eye and outer canthi of both the eyes to monitor vertical and horizontal eye movements (EOG channels) respectively and one electrode was placed on each mastoid. The raw EEG recording was sampled at 512 Hz with online band-pass filtering of .05–.200 Hz. This raw EEG data was stored for every participant for later offline analysis.

### EEG data analysis

Given the current focus on auditory distraction (i.e., the processing of the auditory distractor), we only report the analysis of the auditory-based ERPs. EEGLAB, version 13.2 [55], ERPLAB toolbox, version 5.0 [56] and custom written functions in MATLAB were used to analysed the stored continuous raw EEG data from each participant. Initially, any obvious artifact was removed after visually inspecting the entire EEG data. Then the EEG data was re-referenced to the average of both mastoids. The resultant EEG activity was bandpass filtered (0.1 Hz high pass and 30 Hz low pass; 12 dB per octave roll-off), to eliminate any unwanted frequencies attributable to noise. The EEG data then was divided into bins corresponding to conditions and sounds, i.e., ‘standards W0’, ‘standards W1’, ‘novels W0’ and ‘novels W1’. The filtered and binned data were then epoched into a pre-stimulus period of 100 ms and post stimulus period of 1250 ms. The epoched data was then subject to *runica*, an Independent Component Analysis (ICA) algorithm in EEGLAB to identify and remove eye blinks, horizontal eye movements and other artefacts (facial movements). The ICA algorithm resulted in 64 components and based on the scalp topography, activity power spectrum and activity over trials, the artifactual components were identified and removed from the epoched data. To remove any remaining large artifacts, all epochs with voltages exceeding ±100 μV were excluded from the data. Only approximately 2% of the total trials (sounds) for younger adults and 3% of the total trials for older adults were rejected from the ERP analyses.

All epochs generated by the standard tones were averaged together to produce a standard ERP in W0 and W1 conditions. All epochs generated by the novel distractor sounds were averaged together to produce a novel ERP for W0 and W1 conditions. The difference waves were created by subtracting the standard ERPs from the novel ERPs for each condition across each participant. In the standard ERP waveforms, the N1 and P2 and in difference waveforms, the MMN and the P3a ERP components were identified which were then compared between age groups and across W0 and W1 conditions.

### Measuring the ERPs

The ERPs were measured from F3, Fz, F4, C3, Cz, C4 scalp electrode locations. We focused on these six electrodes when deriving N1, P2, MMN and P3a auditory ERPs given the fronto-central topographical distribution reported widely in auditory research [4, 9, 18, 57, 58]. In the standard ERP waveforms, the N1 was identified as the first clear negative peak between 90 and 120 ms and P2 as a second positive peak between 150 and 200 ms across participants. In the difference waveforms, the MMN was identified as first clear negative deflection between 100 and 200 ms and the P3a was identified as first positive deflection between 200 and 300 ms across both the task conditions and all the participants. The mean amplitude of MMN and P3a components in the difference waveforms and N1 and P2 peaks in the standard waveforms was measured as the mean amplitude of the waveform over a duration of ±25 ms from the peak for each participant from each of the six electrodes. Note, we used the term P3a throughout since in our paradigm the auditory stimuli were to be ignored; a later occurring P3 subcomponent, the P3b, occurs for stimuli that are attended [59]. Another subcomponent of the P3 has also been reported, the novelty-P3, which appears to occur even later (360–450 ms); we do not use this term as there is a current debate about how distinct this subcomponent is from the P3a [60, 61]. The peak latencies were identified as the time-point in the standard waveform with largest respective negative (N1) or positive deflection (P2). In the difference waveform peak latencies were identified with largest respective negative (MMN) or positive (P3a). All peak latencies were measured from the scalp location Fz only. Due to temporal proximity between the onset of visual stimulus and the occurrence of the RON responses (400–600 ms), the visual stimulus potentially masked the RON ERP and was not reported in the current study.

### Data analyses

Mean amplitudes and the peak latencies of N1, MMN and P3a ERP components were subjected to a simple 2 (Task; W0 vs W1) × 2 (Age; older adults vs younger adults) mixed analysis of variance (ANOVA). Reaction times and accuracy of the behavioural responses were determined for visual task following the standard and novel sound presentation.

## Results

### Behavioural measures

#### Response times

To determine if there was less disruptive effect of the distractors on visual task performance in the W1 condition compared to the W0 condition, a 2 (Task; W0 vs W1) × 2 (Distraction type; standards vs novels) by 2 (Age; older adults vs younger adults) mixed repeated ANOVA analyses was conducted; wherever the assumption of sphericity was violated the Greenhouse-Geisser correction was applied. The was a significant effect of Task, i.e., participants had longer response times in the W1 visual task condition (496 ms) compared to the W0 task (465 ms), *F*(1,30) = 15.22, *p* < .001, ŋ_p_^2^ = .34. There was also a significant effect of Age, older adults took significantly longer to respond to the visual task than younger adults (OA: 527 ms, YA: 433 ms), *F*(1,30) = 20.03, *p* < .001, ŋ_p_^2^ = .40. The effect of Distraction Type was not significant, *F*(1,30) = .07, *p* = .79, ŋ_p_^2^ = .002. There was a three-way interaction between Task, Age and Distraction type, *F*(1,30) = 6.432, *p* = .01, ŋ_p_^2^ = .18.

**Fig. 2:**
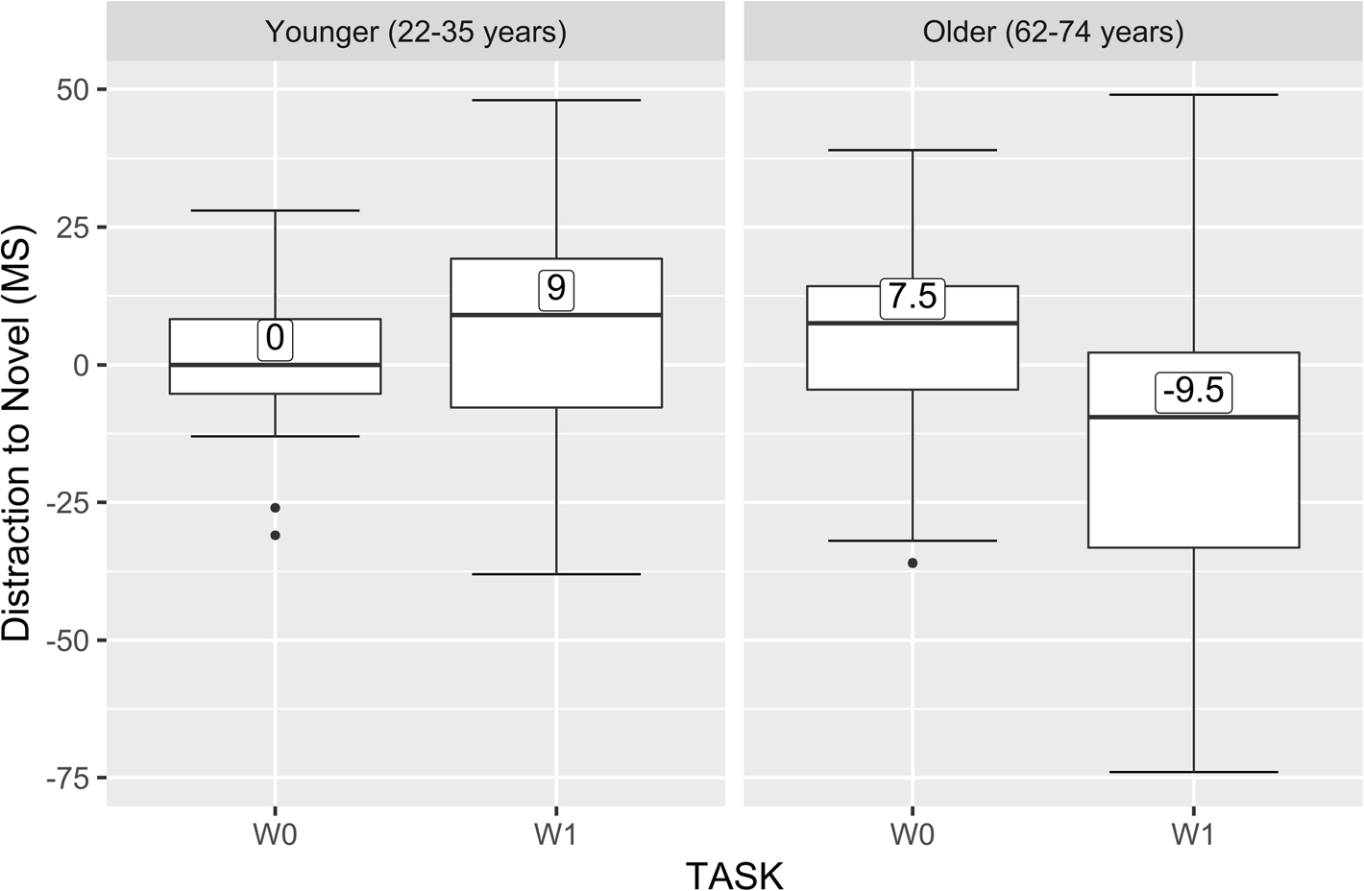
Distraction to the novel sound. Correct response times (ms) for the W0 (no working memory) and W1 (working memory) visual tasks as a function of the presentation of novel minus standard distractor sounds. The left panel shows the older adult data, asterisks indicate the mean, the upper whisker is the largest value no further than 1.5 * IQR; lower whisker is smallest value at most 1.5 * IQR, data beyond the whisker plotted individually. IQR: Inter-quartile range

To index distraction, we calculated the difference in latencies to correctly respond to visual tasks (W0 and W1) when the novel sound was presented minus the correct latencies when the standard sound was presented. These mean difference scores are shown in Fig. 2 for the older and younger adults. A positive score indicates that the correct response times were slower for the novel compared to the standard condition (i.e., distraction due to the novel sound).

As can be seen in Fig. 2, in terms of being distracted by novel compared to standard sounds, older adults were less distracted during the working memory (W1) task (faster correct responses) than during the no-working memory (W0) task, *t*(15) = 3.68, *p* = .002. This effect was in the opposite direction for younger adults, however the difference was not significant *t*(15) = − 0.75, *p* = .46.

#### Accuracy

**Fig. 3:**
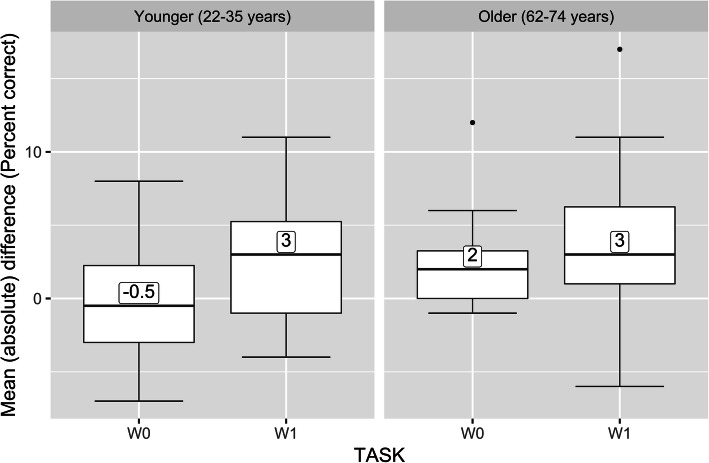
Distraction to the novel sound. Percent accuracy for the W0 (no working memory) and W1 (working memory) visual tasks as a function of the presentation of standard minus the novel distractor sounds. The left panel shows the older adult data

There was a significant effect of Task, i.e., participants were more accurate in the W0 condition (M = 88%) than in the W1 condition (M = 75%), *F*(1,30) = 104.15, *p* < .001, ŋ_p_^2^ = .77. Although the average accuracy score of younger adults was slightly higher (M = 82.9%) than that of the older adults (M = 80.2%), the effect of Age was not significant, *F*(1,30) = .48, *p* = .49, ŋ_p_^2^ = .01. There was a significant effect of distraction type, with the presentation of novel distractors leading to less accurate performance (M = 80.4%) than when standard distractors were presented (M = 82.6%), *F*(1,30) = 12.72, *p* < .001, ŋ_p_^2^ = .30. Once again to index distraction, a difference score was calculated, here it was the difference in percent correct responses to the visual tasks (W0 and W1) when the standard sound was presented minus the percent correct when the novel sound was presented. For this measure, higher scores represent more distraction; mean distraction scores are presented in Fig. 3.

Figure 3 shows that for both older and younger adults, there were more errors in the W1 task (cf., W0) when the novel sound was presented compared to the standard sounds. For older adults this effect was not significant, *t*(15) = − 1.09, *p* = .29; whereas for younger adults it was, *t*(15) = − 2.36, *p* = .03.

### Electrophysiological measures

#### N1-P2 amplitude complex

**Fig. 4:**
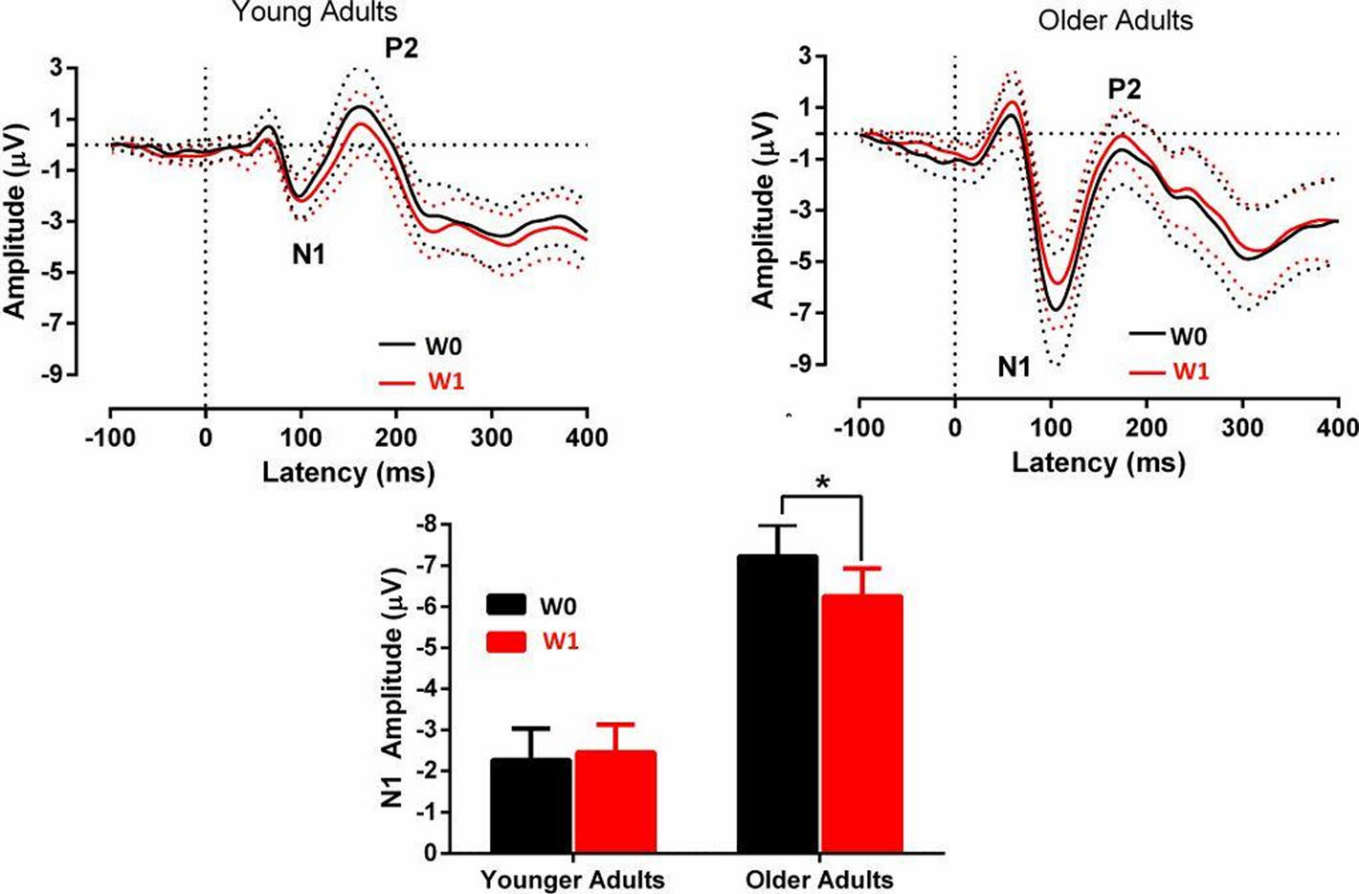
The upper panel represents the grand mean average from the standard ERP and novel ERP waveforms of younger and older adults for the W0 and W1 conditions. The dotted lines indicate 95% confidence intervals across the epoch. The bar-graph in lower panel shows the result of significant interaction between Age and N1 amplitude

Figure 4 (upper panel) shows the grand mean average standard auditory ERPs across W0 and W1 conditions in older and the younger adults and illustrates the N1 and the P2 ERPs.

The mean amplitude of N1 and P2 were separately analyzed using a 2 (‘Task’; W0 vs W1) × 2 (‘Age; older adults vs younger adults) mixed analysis of variance ANOVA. There was a significant difference between the N1 amplitudes of older and younger adults, *F*(1,30) = 16.61, *p* < .001*,* ŋ_p_^2^ = .35; with older adults exhibiting a larger N1 mean amplitude (M = − 4.43 μV) than younger adults (M = − 1.22 μV). The analyses found that the N1 amplitude did not differ significantly across participants between W0 and W1 tasks. There was a significant interaction between Task and Age, *F*(1,30) = 7.83, *p* = .009*,* ŋ_p_^2^ = .20. Analysis of this interaction revealed that the mean N1 amplitude to standards was smaller in older adults during the W1 task, (W0: M = − 4.87 μV; W1: M = − 3.99 μV), *F*(1,30) = 6.82, *p* = .01*,* ŋ_p_^2^ = .18. There was no difference in the N1 amplitude across tasks among younger adults.

There was no effect of Task on the P2 mean amplitude. Overall, older adults (M = − 2.45) had a significantly smaller P2 amplitude than younger adults, (M = .47 μV), *F*(1,30) = 10.22, *p* = *.*003, ŋ_p_^2^ = .25. Also, there was a significant interaction between Task and Age factors, *F*(1,30) = 4.59, *p* = .04*,* ŋ_p_^2^ = .13 with no differences in P2 mean amplitude between W0 and W1 conditions for both older and younger adults.

The peak latencies of N1 and P2 were measured from the frontal electrode Fz and were subjected to same contrast analyses as amplitude. The results did not reveal any significant differences in N1 and P2 latencies for Age and Task factors.

#### MMN

**Fig. 5:**
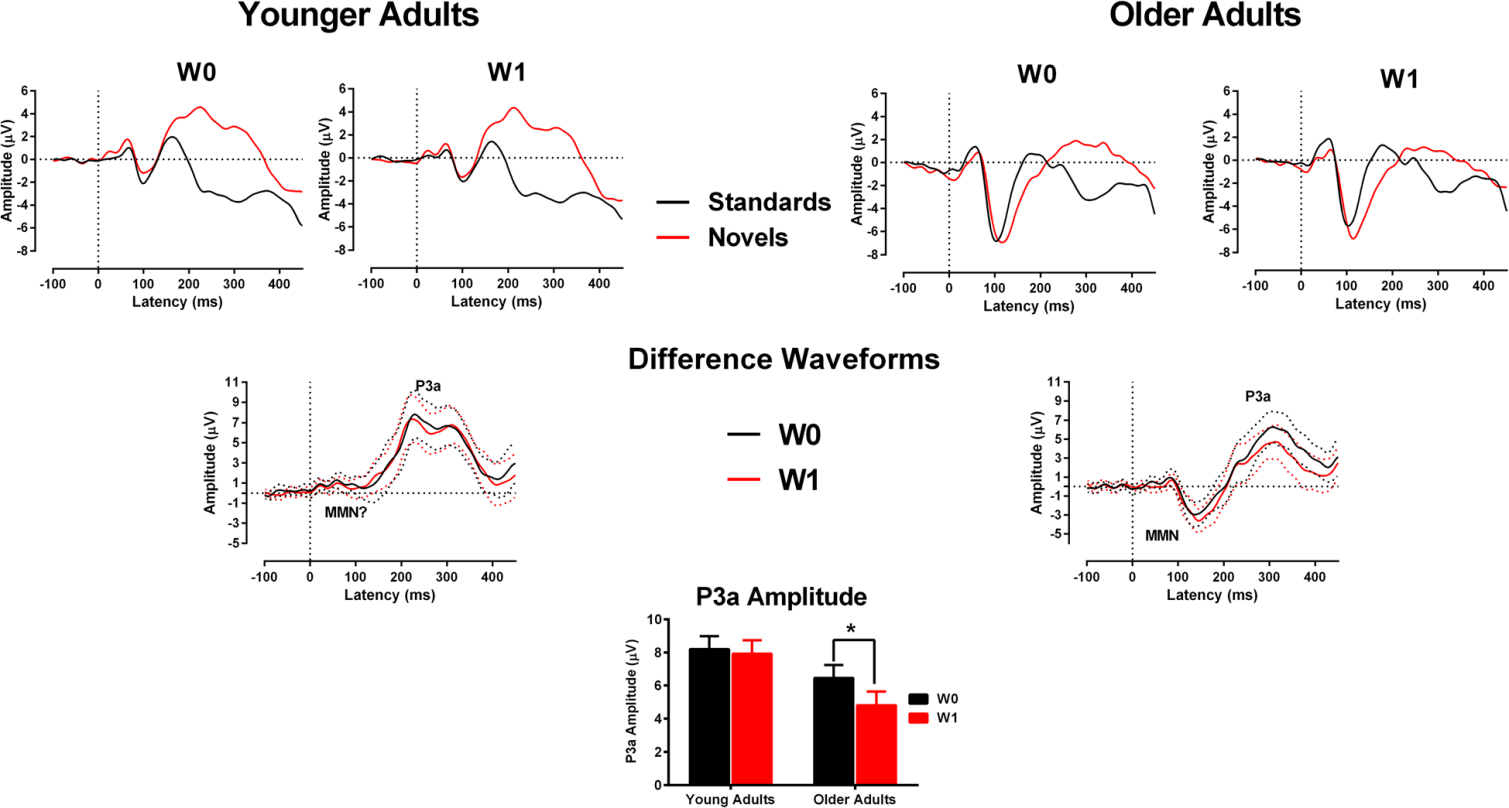
The upper panel represents grand mean average from the standard ERP and novel ERP waveforms of younger and older adults for the W0 and W1 conditions. The middle panel represents the grand mean average from the difference waveforms of younger and older adults for the W0 and W1 conditions. The dotted lines indicate 95% confidence intervals across the epoch for the difference waveforms. The bar-graph in the lowest panel indicates the significant interaction between Age and P3a amplitude

Figure 5 (upper panel) illustrates the grand mean average standard and novel ERPs across tasks for older and younger adults. The middle panel represents difference waveforms obtained by subtracting standard from novel ERPs in older and younger adults across tasks. Both these figures represent averaged ERPs measured from F3, Fz, F4, C3, Cz, C4 scalp electrode locations.

The mean MMN amplitude was analysed only in older adults as no identifiable MMN was observed at the fronto-central electrodes in younger adults (see Fig. 5). The mean MMN amplitude in older adults was analysed using paired-sample t-test between W0 and W1 conditions. The results revealed that the W1 condition did not influence the MMN amplitude in older adults, *t*(1,15) = 1.57, *p* = .13, ŋ_p_^2^ = .14. The MMN latency also did not differ significantly among older adults, *t*(1,15) = −.93, *p = .36,* ŋ_p_^2^ = .05.

#### P3a

The P3a was readily identified in both older and younger groups (fronto-central electrodes). An ANOVA was conducted on the average P3a mean amplitude from six fronto-central electrodes for both groups. Results revealed a significant main effect of Task on the mean P3a amplitude, *F*(1,30) = 10.57, *p* = .003*,* ŋ_p_^2^ = .26, with reduced P3a amplitude in the W1 compared to the W0 condition. The younger adults had significantly larger P3a amplitude than older adults, *F*(1,30) = 4.93, *p* = .03*,* ŋ_p_^2^ = .14 and the interaction between Age and Task was significant *F*(1,30) = 5.36, *p* = .02*,* ŋ_p_^2^ = .15. To investigate this interaction, the mean P3a amplitude for older adults in W1 condition (M = 4.80 μV) was compared to the mean amplitude for W0 condition (M = 6.46 μV); the difference was significant, *F*(1,30) = 15.50, *p* < .001*,* ŋ_p_^2^ = .34. However, in the younger adults, this difference in P3a amplitude between conditions was not significant, see Fig. 5).

The peak latency of P3a was significantly delayed in older adults (M = 301 ms) compared to younger adults, (M = 258 ms), *F*(1,30) = 17.99, *p* < .001, ŋ_p_^2^ = .37 measured from Fz. The task conditions did not modulate P3a latency in either older or younger adults.

In summary, for older adults, the N1 to standard sounds had a reduced amplitude during the W1 compared to the W0 task. In older adults, the size of pre-attentive MMN did not differ between the two task conditions. The size of P3a did not differ between the task conditions in younger adults, whereas the size of the P3a reduced for older adults in the working memory task (W1). Overall, the P3a amplitude was smaller and the P3a latency delayed in older adults.

## Discussion

The aim of this study was to determine whether the effect of auditory distraction would be reduced for older adults when they are engaged in a working memory task compared to a similar task that did not involve working memory. To test this, older adults performed two types of visual task, one that required working memory (W1) and one that did not (W0); distraction consisted of the presentation of a standard tone or a novel sound (younger adults were also tested for comparison). To index the effect of auditory distraction, we used behavioural and electrophysiological measures; below we consider the results of each of these in turn.

### Behavioural distraction effects in older and younger adults

The pattern of the response data suggests that performance on the one-back working memory task (W1) required more cognitive resources than did the no-working memory task (W0), i.e., more errors were made on the W1 task than on the W0 task, and correct responses were slower for the former task. In addition, there was a general effect of age; older adults took longer than younger adults (by about 94 ms) to correctly respond, although they were not more error prone. For older adults, it was found that the deleterious effect of presenting a novel sound on the time taken to correctly classify a visual target was slightly reduced when the classification task required working memory (this protective effect was only shown in response times). Performing the W1 task did not have this effect for the younger adults; distractor type did not influence response times, and when novel distractors were presented compared to standard sounds, younger adults made more errors on the W1 task. Although the current results agree with several previous studies in older adults [23, 62], they are inconsistent with other studies [43, 44] that have reported no age-related behavioural distraction effects. It is noteworthy that, except Tusch et al. [62], task load was not manipulated in any of these studies.

The older adult response time results are consistent with the idea that working memory plays a role in attentional control, such that engaging in a working memory task can reduce distraction from task-irrelevant novel sounds. What is interesting is that there was no evidence of this effect in the younger adult data; the implication being that in performing the W1 task, the younger adults engaged attention to a lesser degree than the older adults. It appears that the younger adults did not try as hard as the older adults, but due to having more efficient attentional processing were able to respond faster than the older adults while maintaining a similar error rate. If this were the case, we would have expected a similar pattern of results in the EEG data (i.e., stronger attentional modulation for the older adults).

### Working memory and N1

The amplitude of the auditory N1 ERP is influenced by bottom-up stimulus properties and also by top-down factors, as such it provides a measure of the extent to which factors like attention can influence relatively early perceptual processing. For example, in the Alain and Woods [44] study, younger and older participants performed a visual task while task-irrelevant tones were presented. They found that the N1 ERPs from older adults were larger than the younger adults and interpreted this as supporting the inhibitory deficit hypothesis of aging (that older adults had deficits in the ability to filter out task-irrelevant stimuli).

We also found that the N1 to task-irrelevant sounds (standard tones) was larger for older adults than younger adults; this was the case for both the W1 and W0 conditions. This result is consistent with previous studies that have reported an increase in N1 amplitude with age irrespective of the listening task (active or passive [63, 64];). There are, however, studies that have reported no change or a decreasing N1 amplitude with age [28, 64–67]. Thus, it is unclear how to interpret this difference, since it could be simply that N1 is larger for older adults regardless of whether the sounds are task-irrelevant or not. Moreover, results from studies that have compared the amplitude of auditory N1 for older and younger adults are inconsistent. Thus, Amenedo & Diaz [64] found that N1 was larger in older compared to younger adults regardless of attention paid to the stimulus, whereas Tusch et al. [62] found that N1 amplitude was greater for ignored than attended sounds.

To test the extent to which older adults have the capacity to filter out task irrelevant stimuli one can examine what happened to N1 amplitude when older adults performed the more attentionally demanding W1 task compared to the W0 task. We found that for older adults, the N1 amplitude to standard sounds was smaller during the working memory task (W1) than in the no working memory task condition (W0). Our results show that older adults can inhibit the task-irrelevant sounds reflected in relatively early evoked response under high cognitive load condition such as working memory.

This result is similar to that from two recent studies [62, 68], that tested younger adults, and found that N1 to task-irrelevant auditory events was smaller under high load. These results suggest that engaging working memory processes can modulate the involuntary sound detection mechanisms indexed by N1. The results with older adults also set a limit on the scope of the inhibitory-deficit hypothesis which proposes that older adults may not be able to inhibit the distractor sounds which may increase the processing of these sounds resulting in an increased N1. That is, the current results show that older adults can inhibit a relatively early evoked response to a distractor sound.

Given that, for younger adults, there appeared to be no behavioural indication of a working memory task reducing the effect of a distracting novel sound, it is perhaps unsurprising that for younger adults’ N1 amplitude did not differ between W1 and W0 conditions. This lack of modulation of N1 amplitude as a function of performing the W1 versus W0 task was also found by SanMiguel et al. [3]. This null result likely reflects the relative ease that younger adults had in performing the current W1 task (which was based on that of SanMiguel et al). Studies with younger adults that have used more difficult tasks (e.g., setting 80% target hit ratio-false alarm ratio) have either shown that N1 amplitude is reduced in the high versus low load condition [62], or with tasks of even greater difficulty, shown that N1 amplitude is increased in the high load condition [5].

### Working memory and MMN

The automatic sound-change detection and auditory discrimination process which is indexed by MMN is often considered to be the first stage of distraction processing in an auditory oddball paradigm [3, 4, 69]. For older adults there was no difference in the amplitude and latency of MMN for W1 and W0 conditions. This suggests that the change detection mechanism was not affected by working memory manipulation and is consistent with the view that to a large extent the MMN is unaffected by top-down information processing [70]. Likewise, this result is in accord with studies (with younger adults) that have investigated the control of initial sound change detection and discrimination mechanisms by executive functions like working memory [3, 4, 71]. It is, of course possible that the 1-back working memory task in the current study was not adequate to modulate the MMN amplitude. Indeed, the study of Lv et al. [5] employed a greater working memory load than the current study and found a larger MMN under a high working memory load. Note, the current study employed a relatively easy working memory task as we tested older participants. Indeed, higher accuracy rates on difficult working memory tasks such as 2-back tasks have been found [72, 73], however, in the current study our aim was to directly compare our results with San Miguel et al’s study and thus we kept the working memory task as a relatively simple 1-back task.

The absence of a reliable MMN in younger adults was unexpected and meant that a direct comparison with older adults could not be made. It is unclear why there was no reliable MMN in younger adults. There seem to be two possible types of explanation for this finding. The first, rather unsatisfying explanation, is that the current cohort of younger adults had unreliable MMNs. That is, studies have suggested that for some individuals an MMN in simple auditory oddball paradigms can be difficult to detect [74–77]; so, it may be that by chance the currently tested younger adults just happened to produce weak MMNs. A second possibility is that the amplitude of younger adult MMN was influenced by the morphology of following P3a. That is, the size of P3a in younger adults was greater and had a more distinct and earlier peak than the older adult P3a; thus if it is presumed that the elicitation of the MMN and the P3a are relatively independent [15], then a large P3a might act to reduce the size of preceding MMN, and this would have occurred to a greater extent in younger adults. Although this is speculative, there have been reports of a dominant auditory ERP peak influencing the morphology of a preceding auditory peak [78].

### Working memory and P3a

The second stage of the distraction processing cycle consists of attention being diverted towards the novel distractor and is indexed by the P3a. It should be noted that while the P3a has been typically associated with distraction and poorer performance, recent studies have identified situations in which an orienting response could facilitate responding, however, unlike the current setup, such situations appear only to apply when the delay between the distractor and the target is greater than 300 ms [79].

We also mentioned above that the P3 ERP is thought to consist of several subcomponents that appear to span slightly different times. It is interesting to note that the evoked P3a for the older adults had a broad distribution that peaked about 301 ms, whereas the P3 for younger adults had a double peak; with the largest amplitude peak peaking at 258 ms. One possibility for why the younger adults showed a double-peaked P3a is the use of salient novel sounds in our paradigm. Escera et al. [12] reported the presence of double-peaked P3a when standard sounds were subtracted from novels and a single-peaked P3a when simple tone deviants were used in an oddball paradigm. Also, we inspected difference waveforms at three parietal electrodes (P3, Pz, P4; see supplementary Figures S1 and S2) to determine if the double peaks in younger adults and the late P3a latency in older adults might be a possible indication of a P3b component. It is clear from figures S1 and S2 that the amplitude of the positive component between 200 and 300 ms reduces posteriorly from frontal to parietal electrodes. The smaller size of P3a at parietal regions and the reduction in amplitude indicate that the positive peak is likely the P3a and not the P3b and suggest that participants were able to ignore task-irrelevant sounds as instructed.

The age-related changes in amplitude and latency of P3a found in the current study are in broad agreement with precious reports [27, 79, 80]; although it is unclear whether these difference in morphology reflect the recruitment of different attentional processes. Nevertheless, in older adults, P3a amplitude was reduced for the W1 compared to the W0 task (whereas it was not for younger adults). It has been suggested that the attenuation of the P3a may be due to the load imposed on working memory by the W1 task preventing the new information from the novel distractor sound being integrated into an on-going model of the auditory environment [3]. It should be noted that, SanMiguel et al. [3] indicated that this argument was speculative and based on the idea of that the effect was mediated by the late phase of the novelty-P3a recorded from parietal electrodes of a double-peaked P3a in younger adults. We however combined the two phases of P3a in younger adults to facilitate comparison with older adults who had only a single peak P3a. In a same vein, Berti & Schroger [4] have suggested that engaging working memory resources provides protection from distraction by attenuating an involuntary shift in attention to the distractor sounds. That is, the deployment of cognitive resources to the primary visual task restricted the available resources for attention switching and distractor processing, resulting in an attenuation of P3a amplitude.

As mentioned, the younger adults did not show a significant reduction in P3a amplitude as a function of performing the working memory task. Although finding no difference in P3a amplitude for younger adults across W1 and W0 conditions is at odds with the results of SanMiguel et al. [3], such fits with the current behavioural results that likewise showed no significant effect of task on distraction by novel stimuli.

To appreciate why ERPs for younger adults may not have been modulated by the working/no working memory task contrast, consider a simple framework that draws on some previous work [81, 82]. Sörqvist and Marsh [81] argue that concentration helps to make people less susceptible to distraction by enabling their locus of attention to become more steadfast. Sörqvist and Rönnberg [82] propose that a high working memory capacity is associated with a more steadfast locus of attention and hence more resistance to distraction. They point out that populations that differ in working memory capacity (e.g., younger, and older adults) will likewise differ in distractibility. Given that distractibility will impact task performance, then if older adults are more distractible than younger adults, they will have to pay relatively more attention to the task to achieve a similar level of performance. One more element is needed to complete this account, i.e., participant motivation to achieve task performance. The idea that participant motivation should be considered in understanding both behavioural and neural performance seems obvious, however, it is only relatively recently that the role of participant motivation has been considered in conjunction with cognitive load in older adults [83].

With the above ideas in hand, a possible reason why the current younger group did not show a clear working memory on distraction effect is that their general performance level (as conditioned by motivation) did not require extensive attentional resources to be expended. That is, younger adults with their relatively high working memory capacity traded-off effort (that would have required increased levels of attention) against task performance. This account has similarities to the task-engagement/distraction trade-off model of Sörqvist and Rönnberg [82], and likewise, in future studies, it would be important to get separate measures of task difficulty and motivation (e.g., self-report). Nevertheless, some support for this general idea comes from a comparison with the overall performance of the younger adults in SanMiguel et al’s study. Compared to the SanMiguel et al’s data, task performance by the younger adults in the current experiment was rather poor, i.e., they made on average approximated three times as many errors and were on average about 23 ms slower in their responses. Indeed, younger adults made significantly more errors when a novel sound was presented in the W1 task compared to the W0 task (something the older adults did not do), indicating that the younger adults’ level of attention was insufficient to avoid being distracted by the novel sounds. Given the absence of an explicit assessment of motivation in the current study, the above explanation for why there were no working memory effects in younger adults is of course speculative.

## Conclusion

In conclusion, the results suggest that engaging attention via a visual working memory task affords older adults some protection from being distracted by a novel sound. This protective effect was shown behaviourally in response times (although not errors); and in evoked auditory potentials by reduced N1 amplitude to standard task-irrelevant sounds and by the attenuation of P3a amplitude to the novel distractors. Younger adults did not show these effects. We proposed that whether such observed effects depend upon the relative difficulty of the task and the participant’s motivation to perform at a high level, i.e., if it is too easy (or they do not try) participants will not need to concentrate. Here, it should be noted that task difficulty is relative; it depends both on the nature of task itself and on the capacity/efficiency of the participant to perform the task (attention/working memory capacity).

## Supplementary Information

**Additional file 1: Figure S1.** The grand mean average difference waveforms of younger adults for W0 and W1 conditions recorded from three frontal, central and parietal electrodes each. The waveforms show a reduction in the amplitude of positive component between 200 and 300 ms from frontal to posterior electrodes, providing no indication of a P3b component.

**Additional file 2: Figure S2.** The grand mean average difference waveforms of older adults for W0 and W1 conditions recorded from three frontal, central and parietal electrodes each. The waveforms show a reduction in the amplitude of positive component between 200 and 300 ms from frontal to posterior electrodes, providing no indication of a P3b component.

**Additional file 3: Table S1.** The ANOVA Statistics of comparisons returned with non-significant results.

## Data Availability

The data used in this study are not available to be shared publicly, since consent to provide and share them was not obtained from participants. But the de-identified data are available from the corresponding author on reasonable request.

## Abbreviations

EEG: Electroencephalography
WM: Working Memory
W0: No working Memory
W1: Working Memory
ERP: Event-related Potential
MMN: Mismatch Negativity
RON: Reorienting Negativity
ANOVA: Analysis of Variance
