# Supplementary material for: Does working memory protect against auditory distraction in older adults?
Source: BMC Geriatr. 2020 Nov 30;20:515. doi: 10.1186/s12877-020-01909-w (PMC7708091; doi:10.1186/s12877-020-01909-w)
Supplement: Supplementary file 3 — Additional file 3: Table S1. The ANOVA Statistics of comparisons returned with non-significant results. [file 12877_2020_1909_MOESM3_ESM.docx]

Table S1: The ANOVA Statistics of comparisons returned with non-significant results.

|  | **Comparison/Analysis** | **F-Ratio** | **p value** |
| --- | --- | --- | --- |
| **N1 Amplitude** |  |  |  |
|  | Task | 0.8 | 0.37 |
|  | Task X Younger adults | 1.81 | 0.18 |
| **N1 Latency** |  |  |  |
|  | Task | 3.44 | 0.07 |
|  | Age | 1.43 | 0.24 |
|  | Task X Age | 1.98 | 0.16 |
| **P2 Amplitude** |  |  |  |
|  | Task | 0.33 | 0.56 |
|  | Younger Adults X Task | 3.71 | 0.06 |
|  | Older Adults X Task | 1.21 | 0.27 |
| **P2 latency** |  |  |  |
|  | Task | 0.03 | 0.86 |
|  | Age | 1.11 | 0.13 |
|  | Task X Age | 0.38 | 0.54 |
| **MMN Amplitude** |  |  |  |
|  | Task (older adults) | 1.57 | 0.13 |
| **MMN Latency** |  |  |  |
|  | Task(older adults) | -0.93 | 0.36 |
| **P3a Amplitude** |  |  |  |
|  | Younger Adults X Task | 0.43 | 0.51 |
| **P3a Latency** |  |  |  |
|  | Task | 0.02 | 0.87 |
|  | Age X Task | 0.06 | 0.8 |
| **Response Times** |  |  |  |
|  | Younger Adults X Task | -0.75 | 0.46 |
| **Accuracy** |  |  |  |
|  | Age | 0.48 | 0.49 |
|  | Older Adults X Task | -1.09 | 0.29 |
